# Cysteine protease of *Clonorchis sinensis* alleviates DSS-induced colitis in mice

**DOI:** 10.1371/journal.pntd.0010774

**Published:** 2022-09-09

**Authors:** Xiaoying Xie, Zhanshuai Wu, Yuhong Wu, Jing Liu, Xinyuan Chen, Xiaoqian Shi, Caiheng Wei, Jiasheng Li, Jiahui Lv, Qing Li, Lili Tang, Shanshan He, Tingzheng Zhan, Zeli Tang

**Affiliations:** 1 School of Pre-clinical Medicine, Guangxi Medical University, Nanning, China; 2 Department of Immunology, Guangxi University of Chinese Medicine, Nanning, China; 3 GuangXi Medical Transformational Key Laboratory of Combine Traditional Chinese and Western Medicine and High Incidence of Infectious Diseases, Nanning, China; 4 Department of Cell Biology and Genetics, School of Pre-clinical Medicine, Guangxi Medical University, Nanning, China; 5 Department of Parasitology, Guangxi Medical University, Nanning, China; 6 Key Laboratory of Longevity and Aging-related Diseases of Chinese Ministry of Education, Guangxi Medical University, Nanning, China; National University of Ireland Galway, IRELAND

## Abstract

**Background:**

Currently, inflammatory bowel disease (IBD) has become a global chronic idiopathic disease with ever-rising morbidity and prevalence. Accumulating evidence supports the IBD-hygiene hypothesis that helminths and their derivatives have potential therapeutic value for IBD. *Clonorchis sinensis* (*C*. *sinensis*) mainly elicit Th2/Treg-dominated immune responses to maintain long-term parasitism in the host. This study aimed to evaluate the therapeutic effects of cysteine protease (*Cs*CP) and adult crude antigen (*Cs*CA) of *C*. *sinensis*, and *C*. *sinensis* (*Cs*) infection on DSS-induced colitis mice.

**Methods:**

BALB/c mice were given 5% DSS daily for 7 days to induce colitis. During this period, mice were treated with r*Cs*CP, *Cs*CA or dexamethasone (DXM) every day, or *Cs* infection which was established in advance. Changes in body weight, disease activity index (DAI), colon lengths, macroscopic scores, histopathological findings, myeloperoxidase (MPO) activity levels, regulatory T cell (Treg) subset levels, colon gene expression levels, serum cytokine levels, and biochemical indexes were measured.

**Results:**

Compared with *Cs* infection, r*Cs*CP and *Cs*CA alleviated the disease activity of acute colitis more significant without causing abnormal blood biochemical indexes. In comparison, r*Cs*CP was superior to *Cs*CA in attenuating colonic pathological symptoms, enhancing the proportion of Treg cells in spleens and mesenteric lymph nodes, and improving the secretion of inflammatory-related cytokines (e.g., IL-2, IL-4, IL-10 and IL-13) in serum. Combined with RNA-seq data, it was revealed that *Cs*CA might up-regulate the genes related to C-type lectin receptor and intestinal mucosal repair related signal pathways (e.g., Cd209d, F13a1 and Cckbr) to reduce colon inflammation and benefit intestinal mucosal repair. Dissimilarly, r*Cs*CP ameliorated colitis mainly through stimulating innate immunity, such as Toll like receptor (TLR) signaling pathway, down-regulating the expression of inflammatory cytokines (e.g., IL-12b, IL-23r and IL-7), thereby restraining the differentiation of Th1/Th17 cells.

**Conclusions:**

Both r*Cs*CP and *Cs*CA showed good therapeutic effects on the treatment of acute colitis, but r*Cs*CP is a better choice. r*Cs*CP is a safe, effective, readily available and promising therapeutic agent against IBD mainly by activating innate immunity and regulating the IL-12/IL-23r axis.

## Author summary

Inflammatory bowel disease (IBD) has become a global disease with accelerating morbidity and prevalence in both adult and pediatric populations, however, options have been limited in the remedy of this disease. IBD-hygiene hypothesis provides a promising strategy for the treatment of IBD. In the present study, we firstly investigated the therapeutic effects of proteins (r*Cs*CP and *Cs*CA) derived from *Clonorchis sinensis* (*C*. *sinensis*) and the parasite itself on DSS induced acute colitis mice. *C*. *sinensis* infection could ameliorate colitis to some extent, but also cause the abnormality of blood biochemical indexes. In contrast, intraperitoneal administration of r*Cs*CP and *Cs*CA achieved better therapeutic effects on DSS-induced acute colitis without causing abnormal blood biochemical indexes. r*Cs*CP was superior to *Cs*CA in alleviating colonic inflammation, enhancing the percentage of Treg cells, and ameliorating types of inflammatory-related cytokines. r*Cs*CP ameliorated colitis mainly through activating TLRs and other related innate immunity, up-regulating the secretion of Th2/Treg related cytokines (e.g., IL-10, IL-4 and IL-13) and down-regulating the expression of inflammatory cytokines (e.g., IL-12b, IL-23r, IL-7 and IL-17A), thereby restricting the transformation of Th1/Th17 cells. Our study suggests that r*Cs*CP is a prospective therapeutic agent for treating IBD. This work also provides novel ideas for the application of parasites and their related products in IBD or other autoimmune diseases.

## Introduction

Inflammatory bowel diseases (IBD), comprising ulcerative colitis (UC) and Crohn’s disease (CD), are chronic idiopathic disorders causing gastrointestinal tract inflammation [1,2]. In the 21st century, IBD has become a global disease with ever-rising morbidity and prevalence in both adult and pediatric populations [1,3]. Presently, anti-inflammatory drugs, antibiotics and biologics, such as salicylates, oral corticosteroids, and monoclonal antibodies, are used to treat IBD. However, these treatment options have problems including long-term medication side effects, easy relapse after drug withdrawal, and high price [2,4,5]. Therefore, there is still a lack of effective therapies for IBD in clinic. New and effective therapeutic strategies for IBD need to be urgently explored.

In 1989, Strachan’s “hygiene hypothesis” posited that exposure to pathogens in early childhood plays a significant protective role against adult infection or reinfection of autoimmune diseases such as hay fever, eczema and asthma [6]. In 2000, Elliott et al. first proposed the IBD-related hygiene hypothesis [7]. He put forward that people in many developed countries who live in increasingly hygienic environments free from exposure to worms will not develop the mucosal Th2 responses stimulated by the parasite and the eggs, making them more susceptible to CD [7]. Later, this hypothesis was supported by numerous researches [8–11]. A growing number of studies demonstrated that parasites and their derivatives such as excretory/secretory products (ESPs) [12], paramyosin (*Ts*Pmy) [13] and a newborn larvae specific serine protease (*Ts*Sp) of *Trichinella spiralis* (*T*. *spiralis*) [14], 16-kDa secreted protein (*Sj*16) [15] and monosexual cercariae [16] of *Schistosoma japonicum* (*S*. *japonicum*), and *Trichuris suis* ova [17], could alleviate experimental colitis and have potential protective effects against IBD.

*Clonorchis sinensis* (*C*. *sinensis*, *Cs*) is also known as liver fluke, and its adult worm can be chronically parasitized in the human bile ducts for a long time, up to 20–25 years, or even lifelong [18,19]. During the parasitism of *C*. *sinensis*, a strong Th1-biased immune response is induced at the young stage of infection, while it switches to a Th2/Treg-dominated immune response at the adult stage [20,21]. Adult crude antigen of *C*. *sinensis* (*Cs*CA) has been documented to elicit Th2-based responses and facilitate the production of cytokines IL-10, TGF-β, IL-13 and IL-4 [22,23]. It has been confirmed that the cysteine protease (CP) superfamily is the most abundant protease in the life history of *C*. *sinensis* [24]. *C*. *sinensis* cysteine protease (*Cs*CP) is expressed in all developmental stages of *C*. *sinensis*, especially in the adult stage [25]. As one of the ESPs of both metacercaria and adults, *Cs*CP plays a critical role in both parasitic and pathological processes of *C*. *sinensis*, such as tissue invasion, immune escape, egg hatching, excystment/encystment [25,26]. Our previous studies revealed that recombinant *Cs*CP (r*Cs*CP), which was prokaryotic cloned by Lv et al, has good immunogenicity and could evoke Th2-dominated and Th1/Th2 mixed immune responses [9,27]. The origin of this *Cs*CP was from a clone (No. *Cs*C61E05) in *C*. *sinensis* cDNA library, which is in the same branch with *Cs*CP3-B5G4x8 and closely related to *Sj*CL-FN316422 with conserved catalytic triad of the Cys/His/Asn [25]. Collectively, *Cs*CP is an important regulatory factor in each growth stage of *C*. *sinensis*, and its function of participating in tissue invasion and immune escape during parasitic infection suggests that *Cs*CP may play a significant role in down-regulating acute inflammation of the host.

In the present study, the therapeutic effects of r*Cs*CP were evaluated and compared with *Cs*CA, *C*. *sinensis* infection and dexamethasone (DXM) on dextran sulfate sodium (DSS)-induced acute ulcerative colitis in mice. In addition, we screened out the key signal pathways of r*Cs*CP and *Cs*CA treatments for colitis to explore the immune molecular mechanisms. Our studied aimed to provide new therapies for IBD and new ideas for the application of parasitic products in the treatment of other inflammatory diseases.

## Methods

### Ethics statement

All the animal research was conducted under the ethical committee for animal experiments of Guangxi Medical University (approval no. 202107003) and carried out following the Guide for the Care and Use of Laboratory Animals of the National Institute of Health in China.

### Animals and parasites

Forty-two female BALB/c mice (4–5 weeks old) were obtained from Hunan SJA Laboratory Animal Co., Ltd. Six female Sprague-Dawley rats (6–8 weeks old) were obtained from the experimental animal center of Guangxi Medical University. All the experimental animals were raised carefully in a temperature-control room (25°C ± 2°C) with a fixed illumination time (12-h light/12-h dark) and standard feed.

Metacercariae of *C*. *sinensis* were derived from *Pseudorasbora parva* that were captured in Hengxian County, Guangxi Province, China. Living metacercariae were collected by routine digestion and isolation (0.2% HCl, 0.6% pepsin, pH2.0) [28].

### Establishment of animal model infected with *C*. *sinensis*

Six rats were infected with living metacercariae of *C*. *sinensis* (180 per rat) by intragastric administration. Eight weeks after infection, the rats were sacrificed to collect adult worms of *C*. *sinensis* from the livers. Similarly, six BALB/c mice were infected with *C*. *sinensis* by intragastric administration. Each mouse was orally infected with 30 living metacercariae. After 35 days of infection, the mice were conducted with follow-up experiments (as the following DSS+*Cs* group).

### Preparation of r*Cs*CP and *Cs*CA protein

r*Cs*CP (accession no. JN655695) was abundantly expressed in strain BL21 (DE3) transformed with the recombinant plasmid of pET-28a (+)-*Cs*CP by isopropyl β-D-thiogalactopyranoside (IPTG) induction. Then, r*Cs*CP was purified with the His Bind Purification kit (Novagen, Darmstadt, Germany) and performed with gradient elution by 5–400 mM imidazole [27].

Several intact and vigorous adults of *C*. *sinensis* were obtained from the bile ducts of rats. After washing the adult worms with PBS, they were cut into pieces, resuspended with an appropriate amount of PBS, and freeze-thawed twice at -20°C. Adult worms crude extract (*Cs*CA) was initially grounded with a glass grinder and then ultrasonically broken twice with a cell crusher (Scientz, Ningbo, China) at 30% amplitude for 15 minutes (ultrasound 1 second interval 2 seconds) each time in an ice bath. Then the supernatant was collected by centrifugation (20 min, at 13,000 rpm and 4°C) and frozen at -80°C.

The endotoxin of r*Cs*CP and *Cs*CA was removed by Detoxi-Gel Endotoxin Removing Gel (Thermo Fisher, Massachusetts, USA) and detected using Rapid Gel Clot LAL Endotoxin Test Kit (Solarbio, Peking, China). The protein concentration was determined by BCA Protein Assay Kit (Beyotime, Shanghai, China). All the protein was sterilized by the 0.22 μm filters before being used in animal experiments.

### DSS-induced colitis and treatment in mice

To induce acute colitis, mice were fed with 5% (wt/vol) DSS (36–50 kDa, MP Biomedicals, Southern California, USA) for 7 consecutive days. DSS solution was prepared with ultrapure water. The mice were divided into the following 7 groups (n = 6 for each group): Water+PBS group, Water+r*Cs*CP group, DSS+PBS group, DSS+r*Cs*CP group, DSS+*Cs* (mice infected with *C*. *sinensis*) group, DSS+*Cs*CA group, and DSS+DXM group. During the experiment, the Water+PBS group and Water+r*Cs*CP group were given ultrapure water every day, and the other groups were given DSS solution. The drinking solution was changed daily. r*Cs*CP, *Cs*CA and DXM were prepared with PBS and filtered with 0.22 μm ultrafiltration membranes (Millipore, Massachusetts, USA).

Recent studies have reported that 50 μg of helminth-derived protein is the usual dose for colitis treatment in mice [29–32]. There are also 20 μg and 100 μg per day or even lower or higher doses [13,31]. So, we chose a moderate dose (50 μg) of r*Cs*CP for therapeutic experiment. 50 μg r*Cs*CP or *Cs*CA per mouse, 20 μg DXM per mouse, or the same volume of PBS (200 μl) was given to mice from the DSS+r*Cs*CP, DSS+*Cs*CA, DSS+DXM, DSS+*Cs* and DSS+PBS groups by intraperitoneal injection daily during DSS-induced colitis, respectively. Concurrently, the same dose of r*Cs*CP or the same volume of PBS was intraperitoneally administrated to the control groups of Water+r*Cs*CP and Water+PBS.

**Fig 1:**
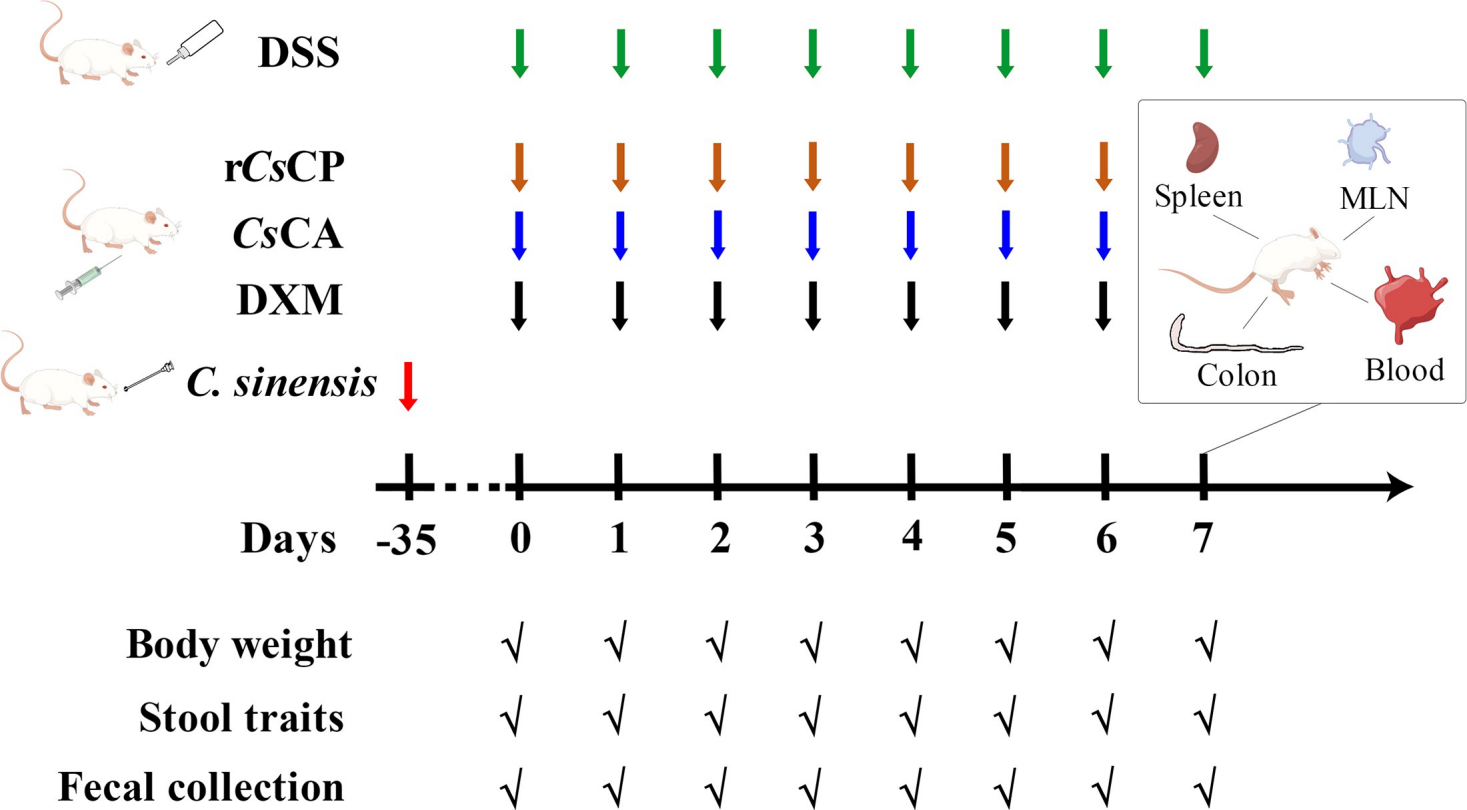
Schematic diagram of DSS-induced colitis and treatment in mice.

From day 0 to day 7, mouse body weight, excrementitious shape, and water intakes were recorded daily. Mouse feces were collected daily for occult blood determination. On day 7, mice were sacrificed to collect blood, which was further used to separate serum and prepare anticoagulated whole blood. Furthermore, the entire colons were separated to record the lengths and evaluate the macroscopic scores. Colon tissue was divided into 3 segments, one for RNA sequencing analysis, another one for histopathological analysis and the last one for myeloperoxidase (MPO) activity measurement. Meanwhile, the spleens and mesenteric lymph nodes (MLNs) of the mice were isolated to prepare single-cell suspensions for flow cytometry. Four samples from each corresponding group were randomly selected for subsequent analysis of blood indicators, pathological damage and transcriptional expression of colon tissue, and the proportion of Treg cells in spleens and MLNs. The modeling, treatment, and sampling collection scheme for acute colitis in mice are presented in Fig 1.

### Clinical scoring of disease

During administration, body weight change, the occurrence of diarrhea and bleeding of each mouse were recorded daily. Fecal occult blood was determined by the Hemoccult assay kit (Jiancheng, Nanjing, China). The total scores of weight loss, diarrhea and bleeding were used as clinical disease scores (disease activity index, DAI), and the scoring criteria are shown in S1 Table [33].

### Macroscopic assessment and histopathological evaluation

After the mice were sacrificed, the entire colon was dissected for macroscopic assessment of tissue damage. The macroscopic score was assessed blindly by an observer unaware of the experimental groupings. Assessment indicators were as follows: hyperemia, wall thickening, ulceration, the spread of inflammation and injury (S2 Table) [34].

Small segments (about 0.5 cm) of colons were fixed with 4% paraformaldehyde, embedded in paraffin, prepared into 5 μm sections (two skip sections per tissue), and then stained with hematoxylin and eosin (H&E) according to standard procedures. Pathological histological assessment of the colon sections was performed blindly as Wang et al. [15], including neutrophil and lymphoid tissue cell infiltration, crypt damage, crypt abscess formation, submucosal edema, goblet cell loss, and reactive epithelial hyperplasia (S3 Table). The histopathological score was the sum of the above parameters.

### Measurement of MPO activity

MPO activity of the infiltrating inflammatory cells (polymorphonuclear neutrophils) in the colon was measured using an MPO assay kit (Jiancheng, Nanjing, China) following the manufacturer’s instructions. MPO activity is expressed as units per kilogram of total protein (U/kg).

### Flow cytometry

To evaluate the proportion of Treg cells, the spleens and MLNs of group Water+PBS, DSS+PBS, DSS+r*Cs*CP and DSS+*Cs*CA were dissected and prepared into single-cell suspensions [35]. Subsequently, cells were incubated with the murine monoclonal antibodies of CD4-FITC, CD25-APC, and Foxp3-PE according to the manufacturer’s instructions of mouse regulatory T cell staining kit (MultiSciences, Hangzhou, China). Finally, the proportion of Treg cells was detected by flow cytometry (BD, Franklin Lakes, USA).

### Multi-cytokine assay

Serum samples were prepared from 4 groups as follows: Water+PBS group, DSS+PBS group, DSS+r*Cs*CP group, and DSS+*Cs*CA group. Cytokines of serum samples were measured using the Mouse Th1/Th2/Th9/Th17/Th22/Treg Cytokine ProcartaPlex Panel (Thermo Fisher, Massachusetts, USA) according to routine multiplex Luminex assay.

### RNA sequencing (RNA-seq) and analysis

To evaluate gene expression levels, total RNA of mouse colon tissue from four groups of Water+PBS, DSS+PBS, DSS+r*Cs*CP and DSS+*Cs*CA were extracted and performed with quantitative and qualitative analysis. Total RNA was sequenced on an Illumina Novaseq 6000 platform by Majorbio Bio-pharm Technology (Shanghai, China). The sequencing data were analyzed on the cloud platform of Majorbio (https://cloud.majorbio.com/), including expression difference statistics, Venn and cluster analysis of differential genes, Kyoto Encyclopedia of Genes and Genomes (KEGG) enrichment analysis, and metabolic pathway analysis.

### Biochemical index detection

On the 7th day of treatment, mice in each group were sacrificed to collect blood samples for preparation of serum and anticoagulated blood. The levels of glutamic pyruvic transaminase (GPT) and glutamic oxaloacetic transaminase (GOT) in the serum were measured using the aspartate aminotransferase assay Kit and the alanine aminotransferase assay Kit (Jiancheng, Nanjing, China), respectively. Blood routine indexes of anticoagulated blood, including white blood cell (WBC), lymphocyte (LYM), monocyte (MONO) and granulocyte (GRAN), were tested using an automatic blood analyzer (Swelab Alfa, Peking, China).

### Statistical analyses

All data were expressed as mean ± SEM (standard error of the mean). One-way analysis of variance (ANOVA) was performed to analyze the significant differences among groups using SPSS 22.0. A *P* value < 0.05 was regarded as statistically significant.

## Results

### Amelioration of clinical activity in DSS-induced acute colitis mice

**Fig 2:**
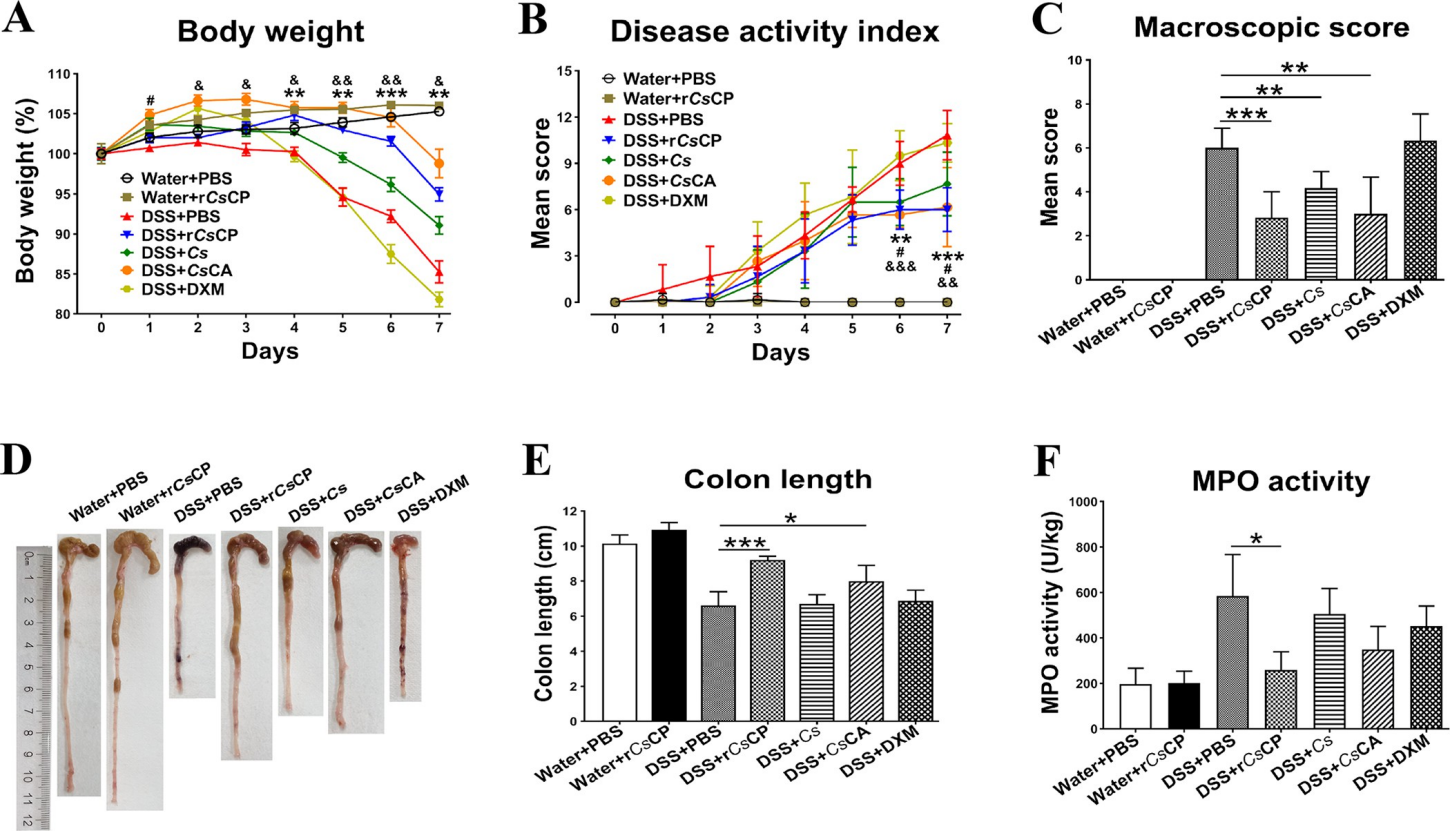
Attenuated clinical activity of DSS-induced colitis in mice by different treatments. (A) The body weight loss of mice in each group for 8 consecutive days (n = 6). (B) The change of disease activity index (DAI) of each group during the trial period (n = 6). (C) The macroscopic score of colons in each group (n = 6). (D) Representative colons from each group. (E) Average length of colon in each group (n = 6). (F) Colonic MPO activity (n = 4). Data are displayed as mean ± SEM. (A) and (B): **P < 0.01, ***P < 0.001 (DSS+PBS group versus DSS+r*Cs*CP group); &*P* < 0.05, &&*P* < 0.01, &&&*P* < 0.001 (DSS+PBS group versus DSS+*Cs*CA group); #*P* < 0.05 (DSS+PBS group versus DSS+*Cs* group). (C), (E) and (F): *P < 0.05, **P < 0.01, ***P < 0.001 (compared with the DSS+PBS group).

To compare the therapeutic effects of r*Cs*CP and *Cs*CA on acute colitis mice, 50 μg r*Cs*CP or *Cs*CA was injected intraperitoneally daily during DSS administration. Results indicated that both r*Cs*CP and *Cs*CA significantly alleviated the weight loss of the DSS-induced mice since 4th and 2nd day of administration, respectively (*P* < 0.05, Fig 2A). Although DSS+*Cs* group also had a certain slowing effect, except for the 1st day, there was no statistical difference on other days (Fig 2A). In addition to weight loss, drinking DSS also triggered colitis symptoms of diarrhea and rectal bleeding in mice. DAI was used to evaluate disease activity clinically. Compared with DSS+PBS group, the cumulative DAI scores of DSS+*Cs*CA, DSS+r*Cs*CP and DSS+*Cs* groups were significantly lower since 6th day of administration (Fig 2B). The average colonic macroscopic score demonstrated that all the r*Cs*CP, *Cs*CA and *Cs* treatment evidently inhibited the increase of DSS-induced colonic gross score (*P* < 0.01), and among them, the improvement effect of r*Cs*CP was the best (Fig 2C).

**Fig 3:**
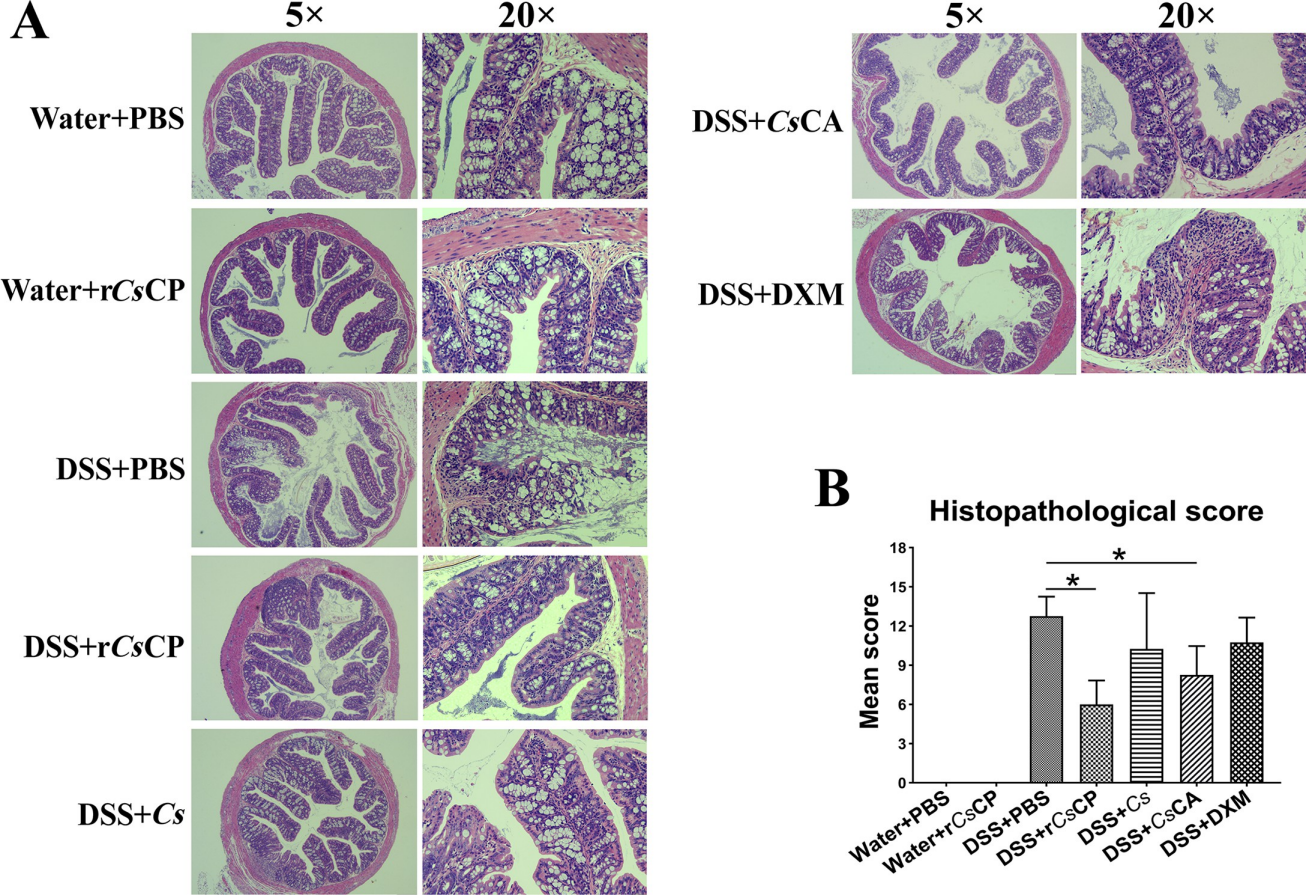
The histopathological changes of colon tissues in each group. On the 7th day of the experiment, the colon tissue of each group mice was isolated, and sections were prepared for H&E staining. (A) Representative colon histopathological sections of each group at objective 5× and 20×. (B) Histopathological scores of colon tissues (n = 4). Mean values ± SEMs are represented by bars. *P < 0.05.

In terms of histomorphological changes, the colon of mice was obviously shortened and intestinal bleeding occurred after DSS induction. Whereas both r*Cs*CP and *Cs*CA treatment could significantly inhibit the shortening and ameliorate severe inflammation-induced hemorrhage (*P* < 0.01, Fig 2D and 2E). Additionally, clinical assessment of disease severity showed that MPO activity was significantly lower in DSS+r*Cs*CP group than in DSS+PBS group (*P* < 0.05, Fig 2F). The histopathological results revealed a prominent pathologic response induced by DSS in colitis mice characterizing severe inflammatory infiltration, intense crypt damage and abscess, depletion of goblet cells, edema, ulcerations, and hyperplasia. Conversely, r*Cs*CP, *Cs*CA, *Cs* and DXM treatment lessened the inflammatory responses and the disruption of colonic architecture (Fig 3). The histopathological scores of groups DSS+r*Cs*CP and DSS+*Cs*CA were significantly lower than those of group DSS+PBS (*P* < 0.05, Fig 3B). Moreover, compared with the Water+PBS group, r*Cs*CP treatment alone did not adversely affect the growth and colonic performance of mice (Figs 2 and 3).

### The proportion of Treg cells population in mice spleens and MLNs

**Fig 4:**
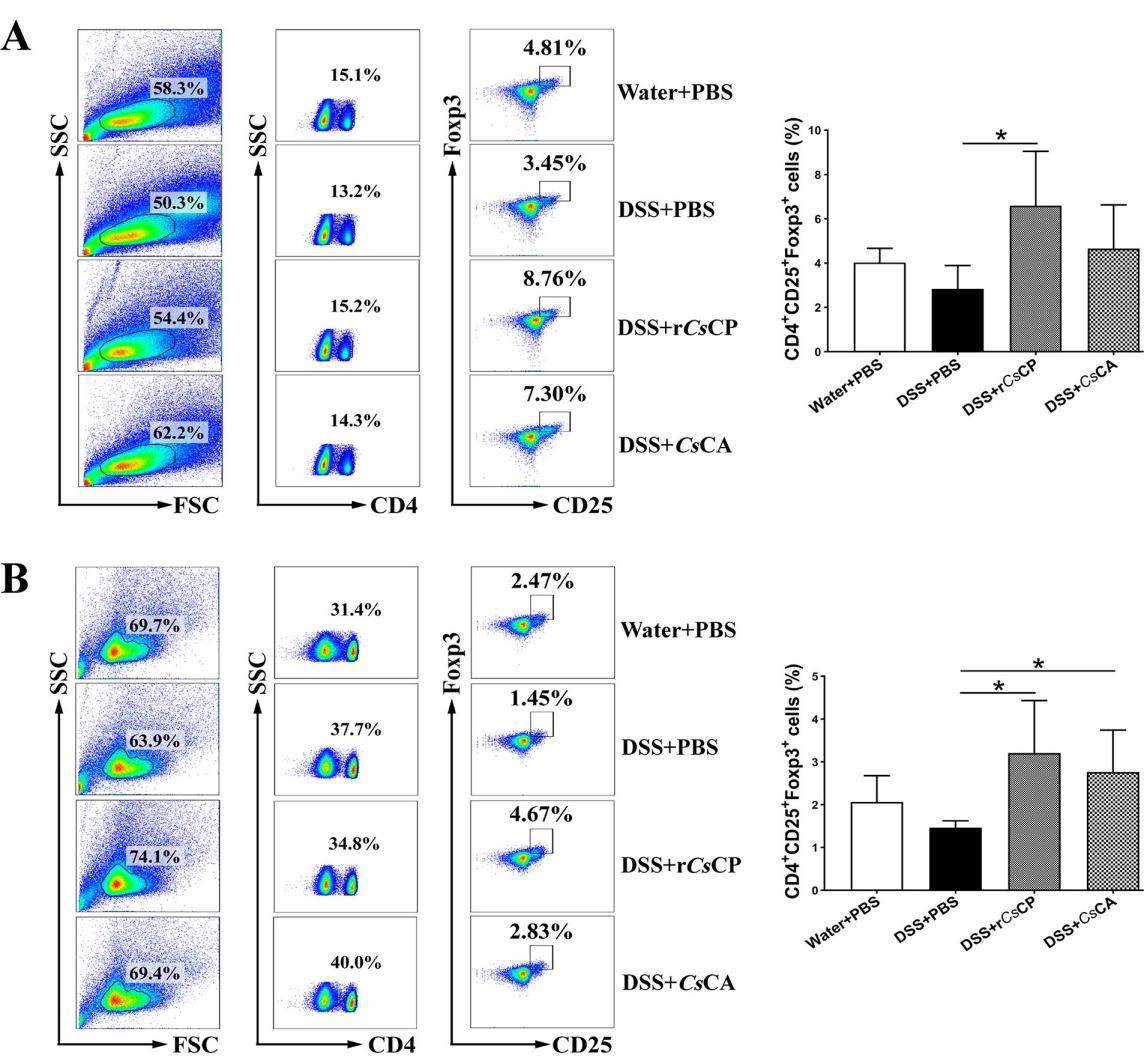
Proportion of CD4^+^CD25^+^Treg cell subsets in mice spleens and MLNs. To detect Tregs, spleens and MLNs were isolated to prepare single cell suspensions for CD4, CD25 and Foxp3 mAbs staining and flow cytometry analysis. (A) Frequency of Treg cells in splenocytes. (B) Frequency of Treg cells in mesenteric lymphocytes. Data are shown as mean ± SEM (n = 4). *P < 0.05.

To evaluate the effects of different treatments on Treg cells in mouse spleens and MLNs, the proportion of the CD4^+^CD25^+^Foxp3^+^ cell population was determined by flow cytometry. The percentages of Treg cells in both spleens and MLNs of r*Cs*CP-treated DSS-induced colitis mice were significantly increased compared with that of PBS-treated DSS-induced colitis mice (*P* < 0.05) (Fig 4). Meanwhile, the *Cs*CA treatment mice also showed a tendency to increase the percentage of Treg cells in both spleens and MLNs, but only caused a statistical increase in MLNs when compared with the DSS+PBS group (*P* < 0.05, Fig 4).

### Cytokine levels in mice serum

**Fig 5:**
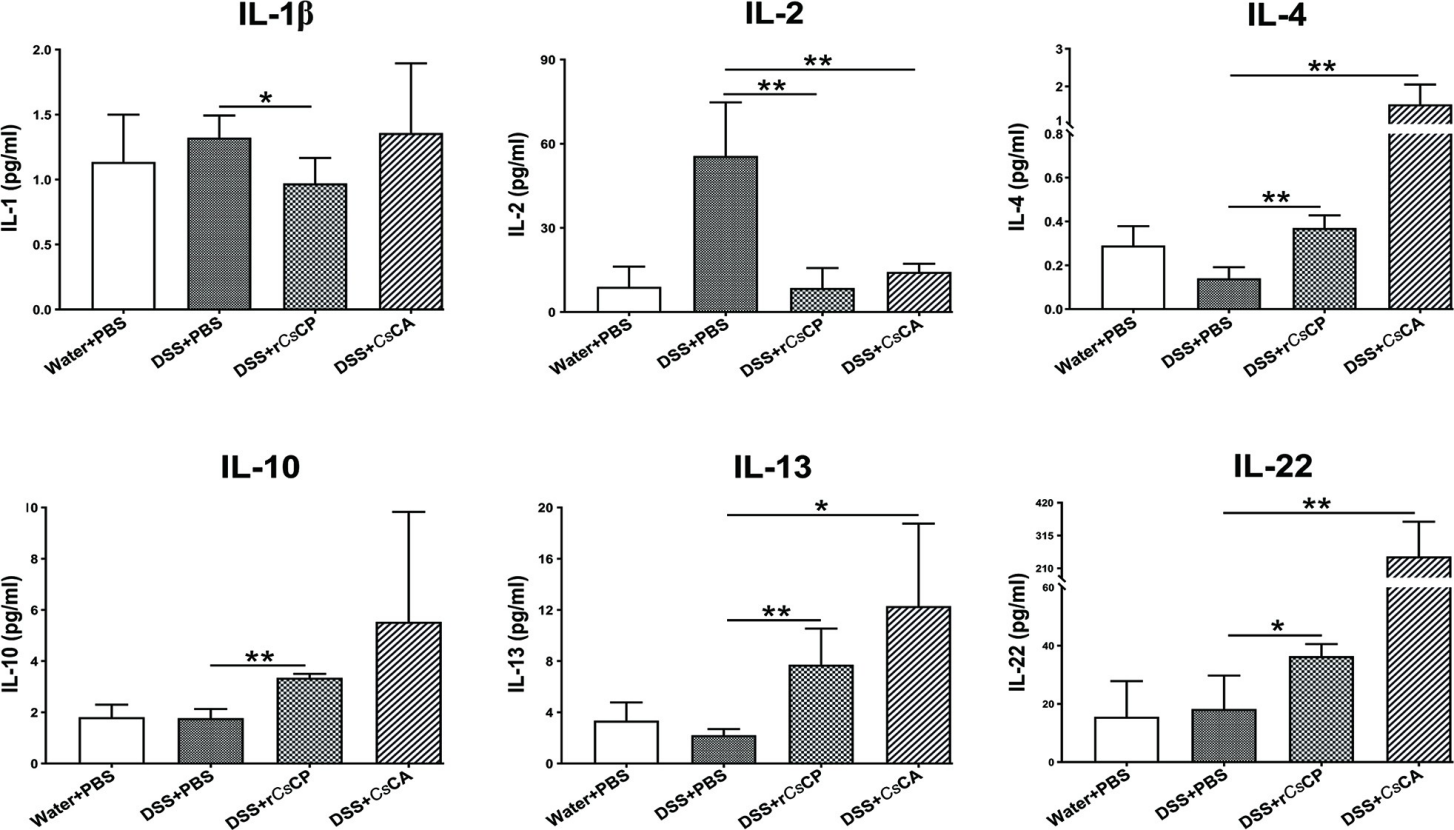
The secretion of inflammatory-related cytokines in serum of different treatments mice. On the 7th day of the experiment, mice were sacrificed to separate serum, and the levels of cytokines, such as IL-1β, IL-2, IL-4, IL-10, IL-13 and IL-22, were measured by ELISA. Data are presented as mean ± SEM (n = 4). *P < 0.05, **P < 0.01.

ProcartaPlex assay results verified that compared with DSS+PBS group, the levels of pro-inflammatory cytokines IL-1β and IL-2 in the serum of DSS+r*Cs*CP group were obviously decreased after r*Cs*CP treatment (*P* < 0.05, Fig 5). On the contrary, the levels of anti-inflammatory cytokines IL-4, IL-10, and IL-13 were significantly increased (*P* < 0.01), and the bidirectional cytokine IL-22 was notably increased in DSS+r*Cs*CP group (*P* < 0.05, Fig 5). Similarly, compared with DSS+PBS group, the IL-2 levels were significantly decreased (*P* < 0.01), the levels of IL-4 and IL-13 were significantly increased (*P* < 0.05), and the IL-22 levels were significantly increased (*P* < 0.01) in the serum of DSS+*Cs*CA group. Additionally, IL-10 levels also increased in DSS-induced colitis mice after *Cs*CA administration, but there was no statistical difference (*P* > 0.05, Fig 5).

### Expression of differential genes with effects on signaling pathways

**Fig 6:**
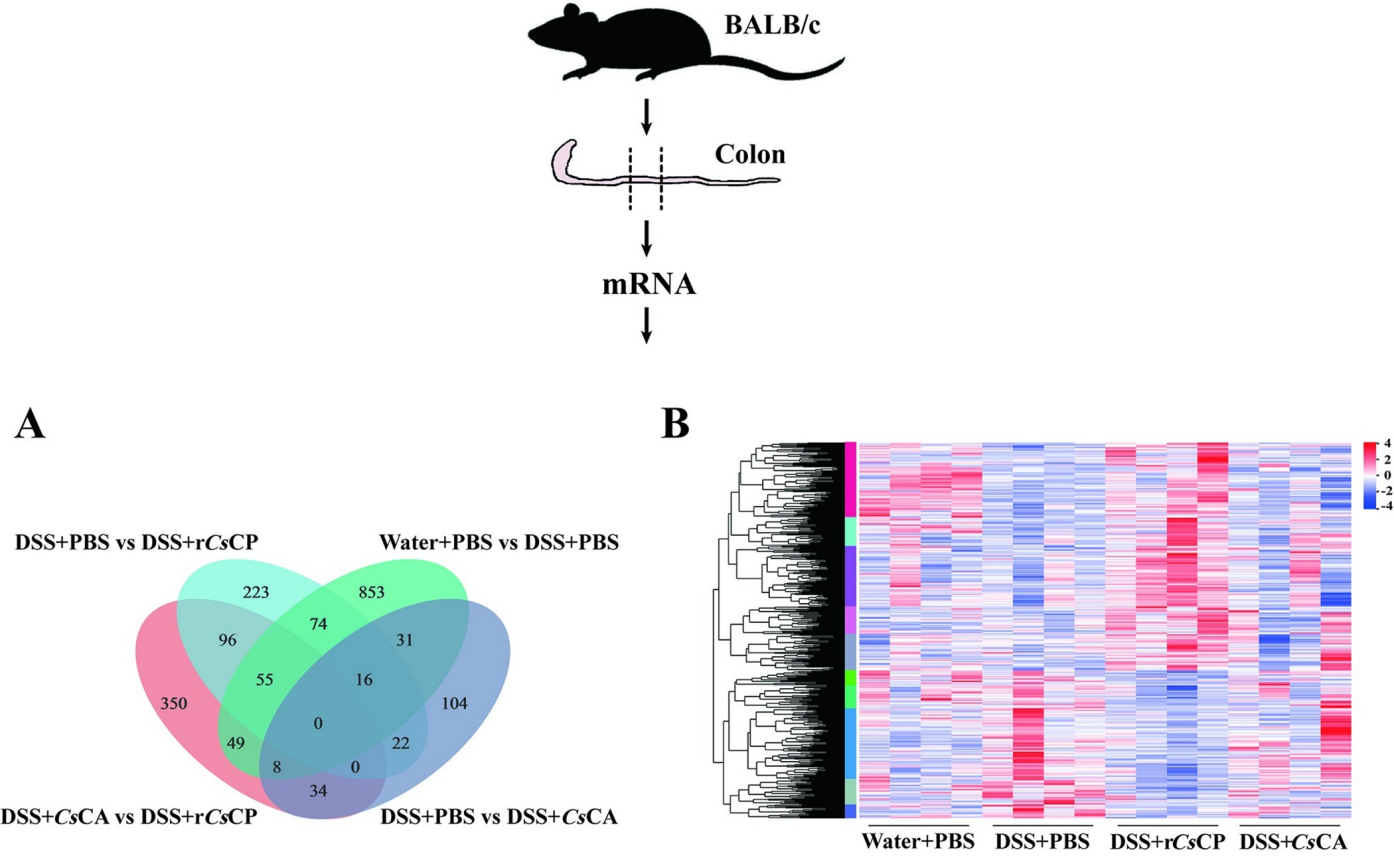
Changes of colonic transcriptome in DSS-induced colitis mice after r*Cs*CP or *Cs*CA treatment (n = 4). (A) Venn diagram of colonic DEGs among four groups of Water+PBS, DSS+PBS, DSS+r*Cs*CP and DSS+*Cs*CA. (B) Heatmap of colonic gene expression levels in four groups. Each column represents a sample, and each row represents a gene. Red and blue indicate gene up- and down-regulation, respectively.

To clarify the differences in colonic gene expression among different treatment groups, transcriptome analysis of the colonic total RNA from Water+PBS, DSS+PBS, DSS+*Cs*CA, and DSS+r*Cs*CP groups was performed. Samples of both r*Cs*CP and *Cs*CA treatments presented good inter-group differences and intra-group reproducibility. Venn analysis and volcano diagrams revealed a total of 486 differentially expressed genes (DEGs) (295 up-regulated and 191 down-regulated) between groups DSS+PBS and DSS+r*Cs*CP, which was more abundant than the 215 DEGs (147 up-regulated and 68 down-regulated) between DSS+PBS and DSS+*Cs*CA (Figs 6A and S1A and S1B). Additionally, 1086 DEGs (472 up-regulated and 614 down-regulated) were differentially expressed between Water+PBS and DSS+PBS groups, and 592 genes (251 up-regulated and 341 down-regulated) were found between groups DSS+r*Cs*CP and DSS+*Cs*CA (Figs 6A and S1C and S1D). Moreover, the DEGs among the Water+PBS, DSS+PBS, DSS+ r*Cs*CP and DSS+*Cs*CA groups were shown as heatmaps in Fig 6B.

**Fig 7:**
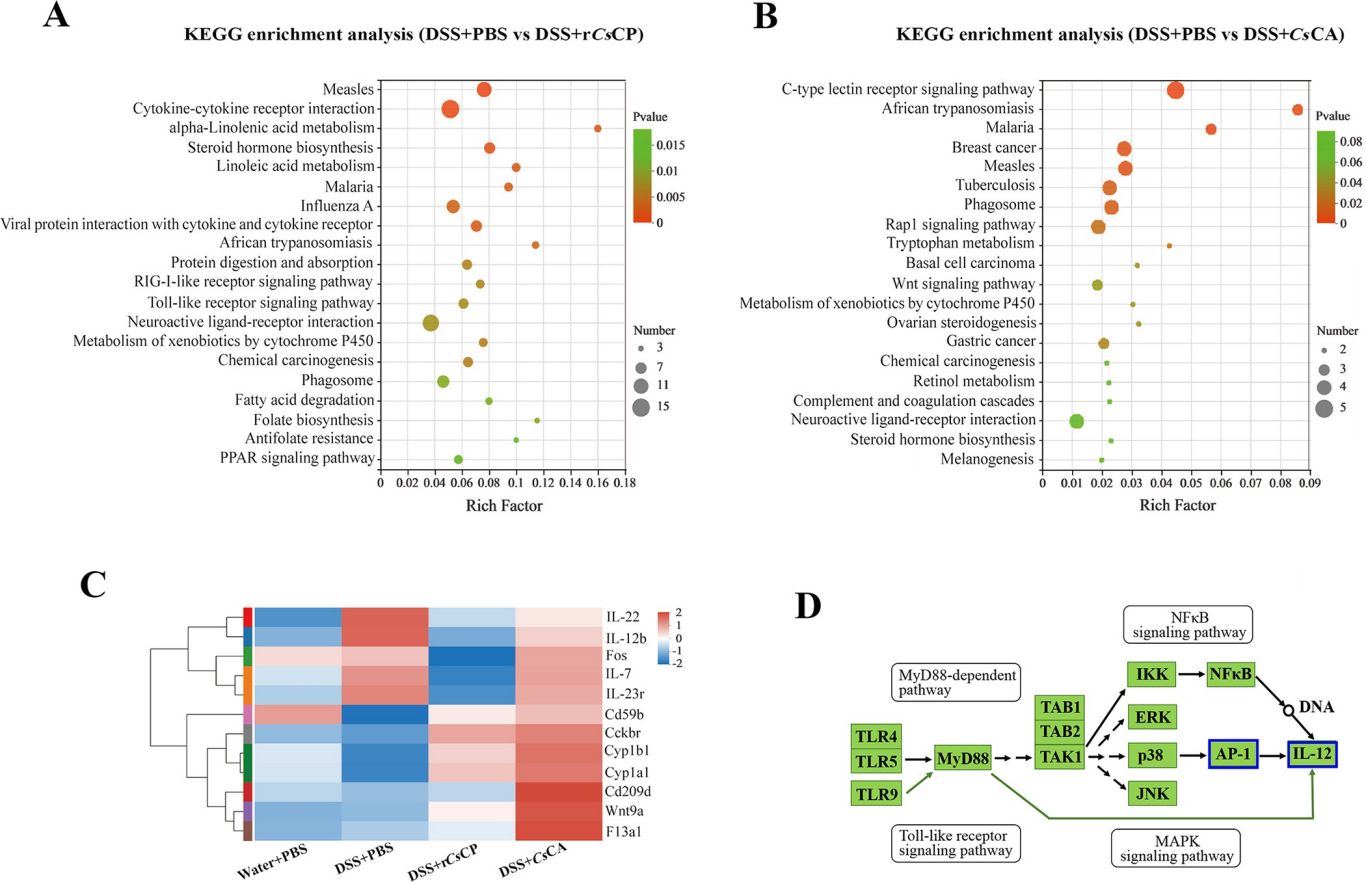
Critical signaling pathways and genes involved in r*Cs*CP- or *Cs*CA- treated DSS-induced colitis mice. (A) KEGG pathway enrichment analysis of the identified DEGs between DSS+PBS and DSS+r*Cs*CP groups. The top 20 most prominently enriched pathways were shown. (B) KEGG pathway enrichment analysis of the identified DEGs between DSS+PBS and DSS+*Cs*CA groups. The top 20 most markedly enriched pathways are shown. (C) Heatmap for the top 12 DEGs involved in most enriched signal pathways after r*Cs*CP or *Cs*CA treatment. Red and blue indicate gene up- and down-regulation, respectively. (D) The mechanism of down-regulation of IL-12 gene in the colon of mice with acute colitis elicited by r*Cs*CP administration. The blue box indicates gene down regulation.

KEGG analysis was used to identify DEGs and the most statistically enriched signaling pathways. The results suggested that immune regulation and metabolism-related signaling pathways, including cytokine-cytokine receptor interaction, Toll-like receptor (TLR) signaling pathway, RIG-I-like receptor signaling pathway, viral protein interaction with cytokine and cytokine receptor, metabolism of xenobiotics by cytochrome P450 and neuroactive ligand-receptor interaction, were highly associated with the mechanism of r*Cs*CP on DSS-induced colitis (Fig 7A). In addition, KEGG analysis of the DEGs between groups DSS+PBS and DSS+*Cs*CA showed that they were mainly concentrated in the C-type lectin receptor (CLR) signaling pathway, Wnt signaling pathway, complement and coagulation cascades, neuroactive ligand-receptor interaction and metabolism of xenobiotics by cytochrome P450 (Fig 7B).

Compared with group DSS+PBS, the expression of IL-22, IL-12b, Fos (AP-1), IL-7 and IL-23r genes in group DSS+r*Cs*CP was significantly down-regulated (Fig 7C and Table 1). Among them, the change of pro-inflammatory factor IL-12 was the most significant, which is downstream of the AP-1 gene (significant down-regulation, *P* < 0.001) and comprehensively regulated by the signal pathways of the TLR, MyD88-dependent, MAPK and NFκB (Fig 7C and 7D and Table 1). The expression levels of Cd209d, Wnt9a and F13a1 genes in group DSS+*Cs*CA were significantly up-regulated than that in group DSS+PBS (Fig 7C and Table 1). The expression levels of Cyp1a1, Cyp1b1 and Cckbr genes in both groups DSS+*Cs*CA and DSS+r*Cs*CP were significantly higher than those in group DSS+PBS (Fig 7C and Table 1). Compared with group DSS+PBS, the Cd59b gene was up-regulated in both group DSS+r*Cs*CP and group DSS+*Cs*CA, but only significantly increased in group DSS+*Cs*CA (Fig 7C and Table 1). RNA-seq data showed that DSS induction increased the transcription levels of IL-17A and IL-17F (significantly up-regulated, *P* < 0.01) in the colon of mice, while the transcription levels of both genes were down-regulated after r*Cs*CP or *Cs*CA treatment, but there was no statistical difference (*P* > 0.05, Table 1).

**Table 1:** Colonic differential gene expression in r*Cs*CP or *Cs*CA-regulated colitis mice.

Gene	Water+PBS vs DSS+PBS	DSS+PBS vs DSS+r*Cs*CP	DSS+PBS vs DSS+*Cs*CA
**IL-22**	↑ (****)	↓ (**)	↓ (ns)
**IL-12b**	↑ (*)	↓ (**)	↓ (ns)
**Fos**	↑ (ns)	↓ (***)	↑ (ns)
**IL-7**	↑ (ns)	↓ (**)	↓ (ns)
**IL-23r**	↑ (ns)	↓ (*)	↓ (ns)
**Cd59b**	↓ (**)	↑ (ns)	↑ (*)
**Cckbr**	↓ (ns)	↑ (*)	↑ (*)
**Cyp1b1**	↓ (*)	↑ (**)	↑ (*)
**Cyp1a1**	↓ (ns)	↑ (**)	↑ (*)
**Cd209d**	↓ (ns)	↑ (ns)	↑ (*)
**Wnt9a**	↓ (ns)	↑ (ns)	↑ (*)
**F13a1**	↑ (ns)	↑ (ns)	↑ (**)
**IL-17A**	↑ (ns)	↓ (ns)	↓ (ns)
**IL-17F**	↑ (**)	↓ (ns)	↓ (ns)

Gene regulation: up-regulation (↑), down-regulation (↓)

Statistical significance

*P < 0.05

**P < 0.01

***P < 0.001

****P < 0.0001

No statistical significance (ns): *P* > 0.05

### Biochemical indexes

**Fig 8:**
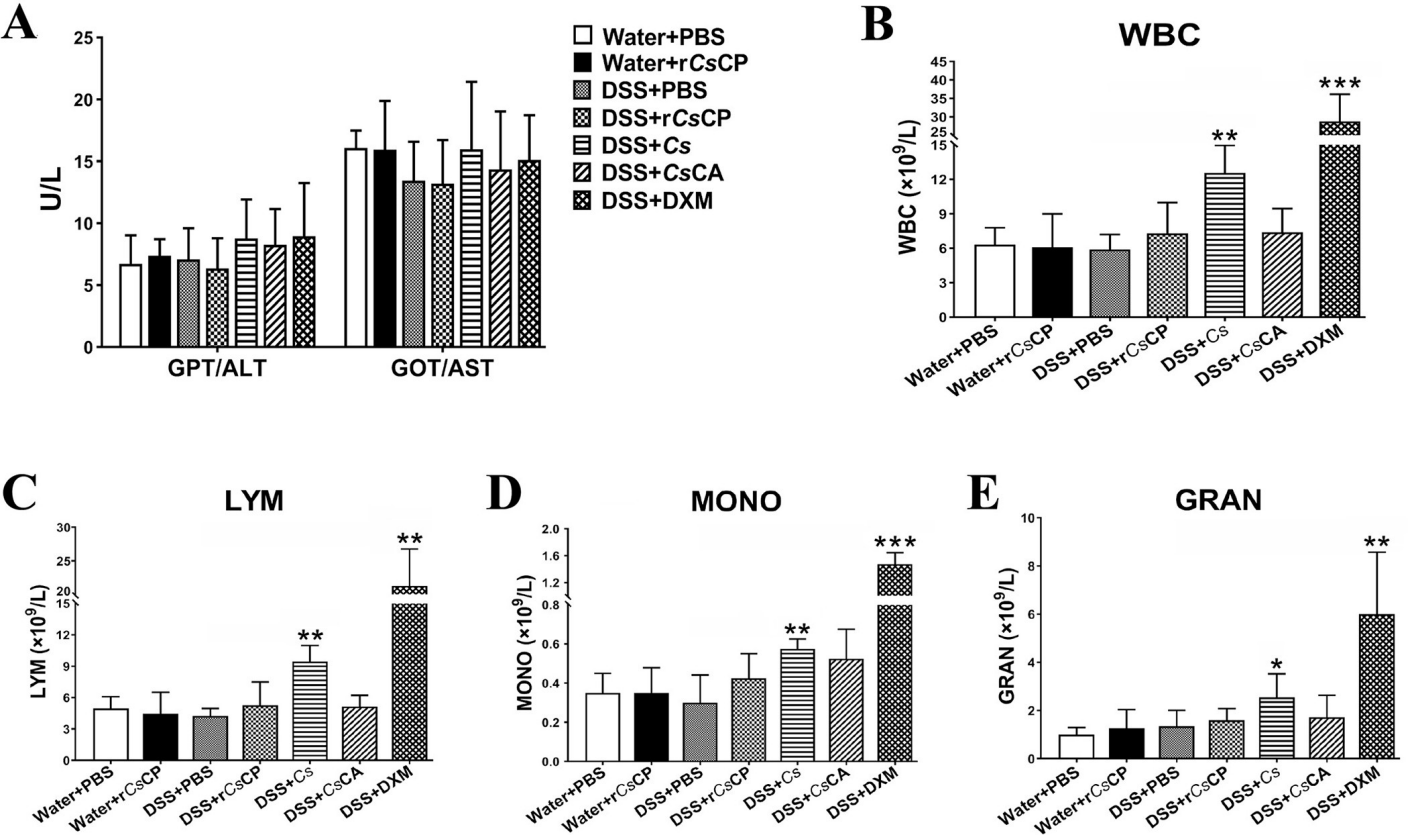
Levels of biochemical enzymes and changes of blood routine indexes. On the 7th day of the experiment, mice were sacrificed to obtain blood, and serum and anticoagulation were prepared for the detection of the activities of GPT/ALT and GOT/AST (A) and the number of WBC (B), LYM (C), MONO (D) and GRAN (E), respectively. Data are displayed as mean ± SEM (n = 4). *P < 0.05, **P < 0.01, ***P < 0.001 (compared with the Water+PBS group).

The levels of GPT/ALT and GOT/AST in the serum of mice among all the seven groups showed no statistical difference (Fig 8A). Blood routine results showed that compared with Water+PBS group, the number of WBC, including LYM, MONO, and GRAN in the anticoagulated blood of mice in groups DSS+r*Cs*CP, DSS+*Cs*, DSS+*Cs*CA, and DSS+DXM were all increased to varying degrees (Fig 8B–8E). All WBC-related indicators in both DSS+*Cs* and DSS+DXM groups increased significantly (*P* < 0.05, Fig 8B–8E). In addition, compared with the Water+PBS group, the levels of WBC, LYM and MONO in the model group of DSS+PBS were decreased (Fig 8B–8E), which may be due to weakened immunity.

## Discussion

In this study, we investigated the therapeutic potentials of *C*. *sinensis* derived proteins r*Cs*CP and *Cs*CA and the parasite itself on DSS induced acute colitis mice. Our data demonstrated that both r*Cs*CP and *Cs*CA treatment, especially r*Cs*CP, effectively alleviated the clinical activity of colitis disease. *C*. *sinensis* infection also attenuated clinical disease activity to a certain extent, but the therapeutic effect was inferior to r*Cs*CP or *Cs*CA. After r*Cs*CP or *Cs*CA treatment, the clinical manifestations, including body weight change and DAI score, were remarkably improved in DSS-induced colitis mice. The colonic pathological inflammation, including macroscopic and microscopic damage, and MPO activity, was significantly alleviated after administration of r*Cs*CP or *Cs*CA. Both r*Cs*CP and *Cs*CA treatment could increase the proportion of Treg cells, up-regulate the levels of anti-inflammatory cytokines, and down-regulate the levels of pro-inflammatory cytokines in DSS-induced mice. Overall, although both were effective, r*Cs*CP treatment showed better therapeutic effects than *Cs*CA treatment as it triggered more abundant differential gene expression and activated more immune-related signaling pathways. All treatments did not cause abnormal levels of GPT/ALT and GOT/AST in serum, indicating that they have no damage to mouse liver [36]. However, blood routine indexes (e.g., WBC, etc.) were abnormally increased in groups DSS+*Cs* and DSS+DXM, and the effect of DXM was not as ideal as that reported by Zhou et al. [16], which are possibly due to the complex mixed immune responses caused by *C*. *sinensis* infection [37], and the different batches and dosage regimens of the drug.

Treg cells play a crucial role in suppressing immune responses and maintaining immune homeostasis in the gut [38]. Decreased Treg cells number and impaired Treg cells function are closely associated with the occurrence of IBD and other inflammatory diseases [39]. Additionally, cytokines play a crucial role in the pathogenesis of IBD [40]. An imbalance in the ratio of proinflammatory and immunoregulatory cytokines derived from regulatory and effector T cells has been verified to induce the development of IBD [40,41]. It has been documented that IL-1/IL-12/IFN-γ axis might be the major pathogenic factor of a subgroup of IBD patients [42]. IL-10 plays an irreplaceable role in coordinating intestinal immune homeostasis, which can be produced by Treg cells [41,43]. Endogenous IL-10 reduces the production of IL-1β and IL-12 by dendritic cells (DCs), thereby constraining the development of Th17 and Th1 cells [42,44]. It has been well established that both IL-13 and IL-4 can down-regulate the secretion of IL-12, hinder the generation of the Th1/Th17 cells, and be closely associated with tissue repair, homeostasis, and maintenance of tissue barriers [45]. IL-2, as a Th1-type cytokine, is critical for maintaining intestinal homeostasis. However, high expression of the IL-2 signaling pathway could predispose to colonic intestinal inflammation [46]. Our current results indicated that r*Cs*CP or *Cs*CA might down-regulate the inflammatory responses of colitis mice via elevating Treg cells in both the spleens and MLNs, inducing the release of Th2 cytokines (IL-10, IL-4 and IL-13), and restraining the production of IL-1β and IL-2 cytokines in serum.

Our RNA-seq results indicated that the therapeutic mechanisms between r*Cs*CP and *Cs*CA were quite different. After r*Cs*CP treatment, more DEGs were produced, and the genes interrelated with cytokine-cytokine receptor interaction and TLR signaling pathway were markedly changed in the colon of colitis mice. IL-12b, IL-7, Fos, IL-23r (IL-23 receptor) and IL-22 genes were significantly down-regulated. IL-17A and IL-17F genes also displayed a downward trend. IL-7 is essential for chronic inflammatory and autoimmune diseases [47]. IL-23, as a member of the IL-12 family, can prompt memory T cells to express the IL-23r, which in turn stimulates the differentiation of Th1 and Th17 cells and the secretion of pro-inflammatory cytokines, e.g., IL-17, IL-6 and IL-22 [48,49]. IL-12, derived from DCs, could direct the differentiation of Th1 and elicit the production of INF-γ [50]. In murine models of colitis, inhibiting the function of IL-12 or IL-23r was confirmed to suppress the inflammatory response dramatically [49]. Therefore, IL-12/IL-23 pathway was identified as a promising target for therapy of human IBD [49]. As the first line of defense, the innate immune system is essential for maintaining intestinal homeostasis and the pathogenesis of IBD [51,52]. Recent studies suggest that IL-23 may be a critical cytokine orchestrated the crosstalk between innate and innate immunity in the pathogenesis of IBD [53,54]. Moreover, our data suggested that the down-regulation of intestinal IL-12 after r*Cs*CP treatment was closely related to innate immunity-related signaling pathways such as TLR, MyD88, NFκB and MAPKs pathways [51,53]. Collectively, we speculated that r*Cs*CP might down-regulate the expression of IL-12/IL-23r through the innate immune system (e.g., TLR signaling pathway), thereby restricting the transformation of Th1 and Th17 cells and inhibiting the production of inflammatory cytokines.

RNA-seq data also indicated the significant up-regulation of Cd209d, Wnt9a and F13a1 genes enriched in pathways related to CLR, Wnt and complement and coagulation cascades signaling pathways might be closely related to the effect of *Cs*CA on DSS-induced colitis. CLRs are transmembrane pattern recognition receptors (PRRs) that may play a crucial role in the progression of IBD [55,56]. SIGNR3 (Cd209d), one of the critical components of CLRs, has been confirmed to alleviate colitis by exerting regulatory signals [57]. Compared with wild-type mice, SIGNR3^−/−^ mice exhibited more severe symptoms of colitis accompanied by a significant increase of TNF-α [55]. The Wnt signaling pathway has been reported to have a prominent role in cell proliferation and tissue repair [58]. However, overactivation of the Wnt pathway has oncogenic potential (e.g., promoting hepatocarcinogenesis) [59] Therefore, the biosafety of *Cs*CA needs to be further studied. Furthermore, coagulation factor XIII (F13), as a connective tissue factor, is conducive to intestinal and mucosal healing [60,61]. It is reported that FXIII-A subunit (F13a) gene deficiency resulted in severe damage to the colonic mucosa in mice with induced colitis, whereas administration of exogenous rF13a significantly alleviated the symptoms [61]. Consequently, it is estimated that *Cs*CA might ameliorate colitis by activating CLR-mediated innate immune responses and initiating pathways related to intestinal tissue and mucosal damage repair.

Furthermore, our RNA-seq results also indicated that both r*Cs*CP and *Cs*CA treatments up-regulated the expression levels of Cckbr and Cd59b genes in the colon, as well as the expression levels of Cyp1 (a and b) genes that are associated with cell metabolism and homeostasis [62]. Studies suggested that Cckbr (also known as CCK2R) participates in the proliferation of colon cells [63], and the protectin CD59 (orthologous to muridae Cd59b) of the complement system could protect the intestinal epithelium from destruction [64]. IL-22 has been reported to be a bifacial cytokine in intestinal inflammation, which is not only conducive to mucosal healing, but also increases the risk of colon cancer if overactivated [65]. Our study revealed that the secretion level of IL-22 was increased in the serum of r*Cs*CP and *Cs*CA-treated colitis mice, whereas the transcription level in the colon was decreased. Compared with the Water+PBS group, the transcription level of IL-22 was significantly increased in the colon of the DSS+PBS group mice. Therefore, we speculated that down-regulation of IL-22 was the strategy by r*Cs*CP and *Cs*CA to prevent severe consequences. However, the specific mechanism needs to be further explored.

Currently, accumulating helminths and their derivatives have been used in attempt to treat IBD in animal models [9,66]. However, different parasites and parasite-derived molecules have different mechanisms for treating colitis. For example, the *T*. *spiralis* derived r*Ts*PMY protein stimulates the expansion of thymic-derived Tregs to maintain intestinal immune homeostasis during inflammation [13]. A 49-kDa r*Ts*Sp molecule modulates the inflammatory process of IBD by inhibiting macrophage infiltration as well as inducing the expression of IL-10 and reducing the secretion of TNF-α [14]. The rSi16 molecule of *S*. *japonicum* protects against colitis mainly through restraining the PPAR-α signaling pathway [15], whereas, the monosexual cercariae treats colitis mostly by switching Th1/Th2 balance and regulating intestinal flora [16]. So far, there are only two reports on the therapeutic effect of *C*. *sinensis*-related molecules on colitis [67,68]. Jang et al. conformed that the recombinant *C*. *sinensis* type I cystatin could alleviate colitis via IL-10^+^F4/80^+^ macrophages recruitment [67]. Hua et al. reported that molecular chaperone HscB protein of *C*. *sinensis* could regulate CD4^+^/CD8^+^T cells balance and inhibit the release of pro-inflammatory cytokines through the MAPK pathway to against chronic ulcerative colitis [68]. Our study suggested that either r*Cs*CP or *Cs*CA might protect against acute colitis by initiating innate immunity and regulating anti-inflammatory adaptive immunity. In addition, we also evaluated the safety of administration of r*Cs*CP and *Cs*CA.

In conclusion, compared with *C*. *sinensis* infection, r*Cs*CP and *Cs*CA were safer and better therapeutic effect on colitis. Comparatively, r*Cs*CP was superior to *Cs*CA in improving colitis inflammation. *Cs*CA alleviated colitis might by regulating CLR-mediated innate immunity and stimulating repair system of intestinal tissue and mucosa. Whereas, r*Cs*CP ameliorated the acute colitis might through activating innate immunity, downregulating the expression of pro-inflammatory factors (IL-12, IL-23r and IL-7), and promoting the production of anti-inflammatory factors (IL-10, IL-4 and IL-13). Overall, r*Cs*CP is an economical and easily mass-produced protein, which is expected to provide prospective therapeutic agent and new ideas for treating human IBD.

## Supporting information

S1 Table Assessment of the DAI. (DOCX)

S2 Table Evaluation of macroscopic scores. (DOCX)

S3 Table Assessment of histopathological scores. (DOCX)

S1 Fig Volcano diagrams of mRNAs comparative gene expression analysis. (A) 486 differentially expressed genes (DEGs) (295 up and 191 down) were discovered between DSS+PBS group and DSS+r*Cs*CP group. (B) 215 DEGs (147 up and 68 down) were discovered between DSS+PBS group and DSS+*Cs*CA group. (C) 1086 DEGs (472 up and 614 down) were discovered between Water+PBS group and DSS+PBS group. (D) 592 DEGs (251 up and 341 down) were discovered between DSS+*Cs*CA group and DSS+r*Cs*CP group.
(TIF)

S1 Data Excel spreadsheet containing, in separate sheets, the underlying numerical data and statistical analysis for Fig panels 2A, 2B, 2C, 2E, 2F, 3B, 4A, 4B, 5, 8A, 8B, 8C, 8D and 8E. (XLSX)
