# Supplementary material for: Cysteine protease of Clonorchis sinensis alleviates DSS-induced colitis in mice
Source: PLoS Negl Trop Dis. 2022 Sep 9;16(9):e0010774. doi: 10.1371/journal.pntd.0010774 (PMC9491586; doi:10.1371/journal.pntd.0010774)
Supplement: S1 Table — (DOCX) [file pntd.0010774.s001.docx]

**S1 Table. Assessment of the DAI**

| Score value | Body weight scores (% body weight loss) | Stool traits | Bleeding |
| --- | --- | --- | --- |
| 0 | < 2% | Normal | No rectal bleeding |
| 1 | ≥ 2 - < 5% | Softer stool/sticks to cage wall | Weak hemoccult |
| 2 | ≥ 5 - < 10% | Moderate diarrhea/unformed stool | Visual blood in stool |
| 3 | ≥ 10 - <15% | Diarrhea (watery stool) | Fresh rectal bleeding |
| 4 | ≥ 15% | - | - |
