# Supplementary material for: Cysteine protease of Clonorchis sinensis alleviates DSS-induced colitis in mice
Source: PLoS Negl Trop Dis. 2022 Sep 9;16(9):e0010774. doi: 10.1371/journal.pntd.0010774 (PMC9491586; doi:10.1371/journal.pntd.0010774)
Supplement: S2 Table — (DOCX) [file pntd.0010774.s002.docx]

**S2 Table. Evaluation of macroscopic scores**

| Colon damage | Score |
| --- | --- |
| No damage, no inflammation and ulcers | 0 |
| Hyperemia without ulcers, smooth surface | 1 |
| Hyperemia and wall thickening without ulcers | 2 |
| One ulceration site without wall thickening | 3 |
| Two or more ulceration sites | 4 |
| 0.5 cm extension of inflammation or major damage | 5 |
| 1 cm extension of inflammation or severe damage | 6-10 |
