# Supplementary material for: Cysteine protease of Clonorchis sinensis alleviates DSS-induced colitis in mice
Source: PLoS Negl Trop Dis. 2022 Sep 9;16(9):e0010774. doi: 10.1371/journal.pntd.0010774 (PMC9491586; doi:10.1371/journal.pntd.0010774)
Supplement: S3 Table — (DOCX) [file pntd.0010774.s003.docx]

## ­S3 Table. Assessment of histopathological scores

| Score | Extent of inflammation | Neutrophil/lymphohi-stiocytic infiltration | Extent of crypt damage | Crypt abscesses | Submucosal oedema | Loss of goblet cells | Reactive epithelial hyperplasia |
| --- | --- | --- | --- | --- | --- | --- | --- |
| 0 | None | None | None | None | None | None | None |
| 1 | Mucosa | Focal | Basal one third | Focal | Focal | Focal | Focal |
| 2 | Mucosa+submucosa | Multifocal | Basal two thirds | Multifocal | Multifocal | Multifocal | Multifocal |
| 3 | Mucosa+submucosa+muscle layer | Diffuse | Entire crypt  damage | Diffuse | Diffuse | Diffuse | Diffuse |
| 4 | Transmura |  | Crypt damage+ulceration |  |  |  |  |
