# Supplementary figures and images for: Cysteine protease of Clonorchis sinensis alleviates DSS-induced colitis in mice
Source: PLoS Negl Trop Dis. 2022 Sep 9;16(9):e0010774. doi: 10.1371/journal.pntd.0010774 (PMC9491586; doi:10.1371/journal.pntd.0010774)

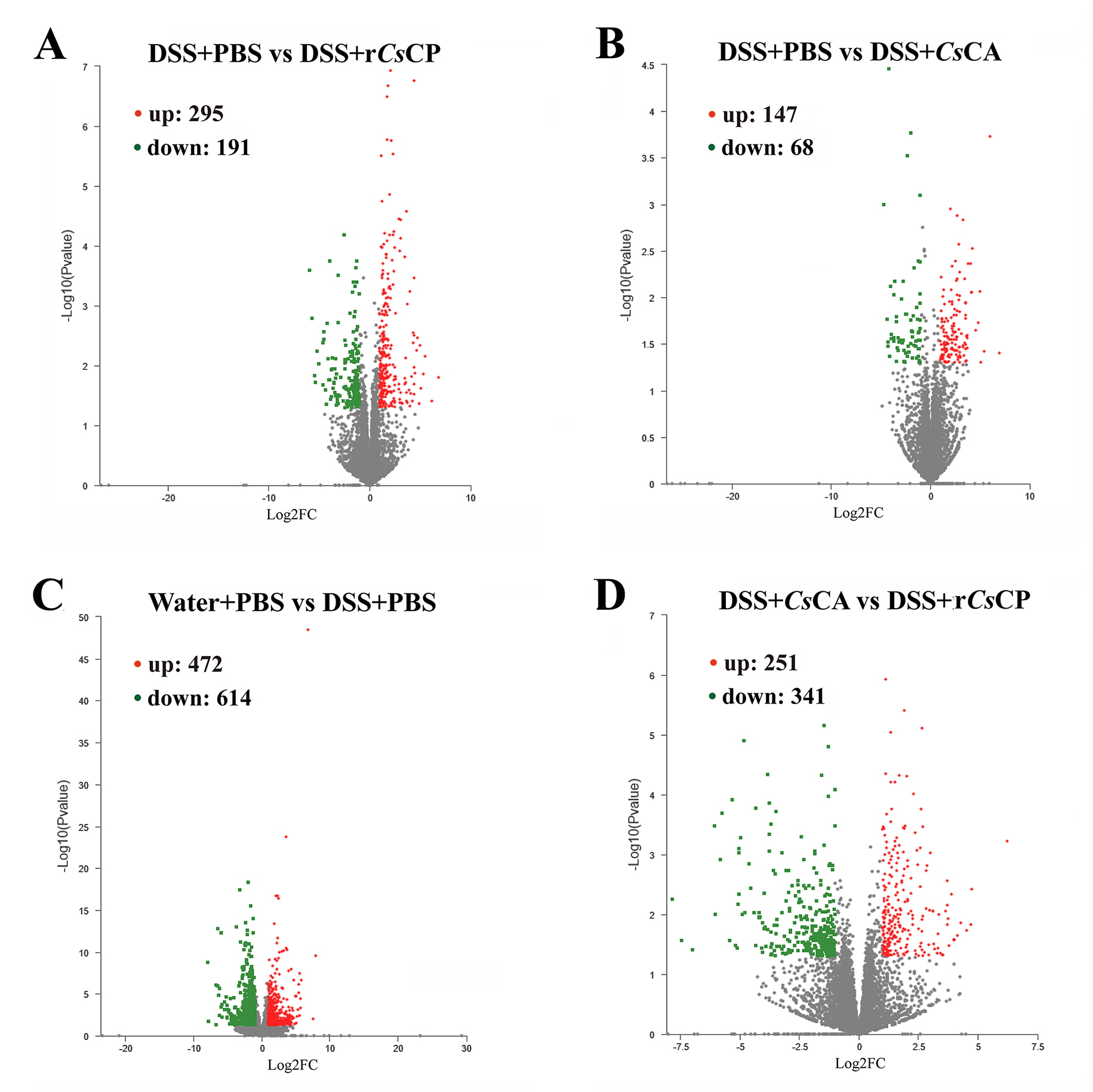

Supplement: S1 Fig — (A) 486 differentially expressed genes (DEGs) (295 up and 191 down) were discovered between DSS+PBS group and DSS+rCsCP group. (B) 215 DEGs (147 up and 68 down) were discovered between DSS+PBS group and DSS+CsCA group. (C) 1086 DEGs (472 up and 614 down) were discovered between Water+PBS group and DSS+PBS group. (D) 592 DEGs (251 up and 341 down) were discovered between DSS+CsCA group and DSS+rCsCP group. (TIF) [file pntd.0010774.s004.tif]
